# A novel ultrasound technique to detect early chronic kidney disease

**DOI:** 10.12688/f1000research.14221.2

**Published:** 2019-02-24

**Authors:** Dulitha K. Hewadikaram, Mudhitha Bandara, Amal N. Pattivedana, Hiran H. E. Jayaweera, Kithsiri M. Jayananda, W. A. Monica Madhavi, Aruna Pallewatte, Channa Jayasumana, Sisira Siribaddana, Janaka P. Wansapura

**Affiliations:** 1 General Sir John Kotelawala Defence University, Rathmalana, Sri Lanka; 2 University of Colombo, Colombo, Sri Lanka; 3 National Hospital of Sri Lanka, Colombo, Sri Lanka; 4 Rajarata University of Sri Lanka, Anuradhapura, Sri Lanka

**Keywords:** Chronic Kidney Disease of unknown etiology, Ultrasound spectral characteristics, Kidney fibrosis

## Abstract

Chronic kidney disease (CKD) of unknown etiology is recognized as a major public health challenge and a leading cause of morbidity and mortality in the dry zone in Sri Lanka. CKD is asymptomatic and are diagnosed only in late stages. Evidence points to strong correlation between progression of CKD and kidney fibrosis. Several biochemical markers of renal fibrosis have been associated with progression of CKD. However, no marker is able to predict CKD consistently and accurately before being detected with traditional clinical tests (serum creatinine, and cystatin C, urine albumin or protein, and ultrasound scanning).

In this paper, we hypothesize that fibrosis in the kidney, and therefore the severity of the disease, is reflected in the frequency spectrum of the scattered ultrasound from the kidney. We present a design of a simple ultrasound system, and a set of clinical and laboratory studies to identify spectral characteristics of the scattered ultrasound wave from the kidney that correlates with CKD. We believe that spectral parameters identified in these studies can be used to detect and stratify CKD at an earlier stage than what is possible with current markers of CKD.

## Introduction

Chronic kidney disease (CKD) is a major public health challenge and a leading cause of morbidity and mortality ^1^. About 8 – 16% of the world population is affected by CKD ^2– 4^ with increased risk for end-stage renal disease, cardiovascular disease, and death ^5^. To date, no specific treatment has shown to arrest the progression of CKD, except dialysis or kidney transplantation ^1^. Considering the high cost of renal replacement therapy, the growing prevalence of CKD has implications for health and social care systems ^6^ especially for developing nations. New variety of CKD has been identified among paddy farmers (known as CKD of unknown etiology (CKDu) or Chronic Interstitial Nephropathy among Agricultural Communities (CINAC)) in the North Central Province of Sri Lanka ^7^. One fifth of the population in Anuradhapura, Polonnaruwa and Badulla districts suffer from CKDu and it has already become a major public health issue in Sri Lanka ^8^

CKD is silent killer because it starts insidiously and progresses slowly until end stage renal disease. The main challenges to improve outcomes in patients with CKD are the inability to identify patients with CKD in early subclinical stages ^9– 11^.

Evidence suggests kidney fibrosis occurs in every type of CKD and leads to progressive and irreversible loss of renal function ^12, 13^. Progressive deposition of extracellular matrix in glomeruli (glomerulosclerosis) and/or interstitial space (tubulointerstitial fibrosis) is known as kidney fibrosis. Recent studies have hypothesized several biomarkers of renal fibrosis. Among them are, transforming growth factor-β1 (TGF-β1) a pro-fibrotic cytokine measured in urine and serum, bone morphogenetic protein-7 (BMP-7) recognized as a natural antagonist to TGF-β1 measured in serum and epidermal growth factor (EGF), a tubule-specific protein critical for cell differentiation and regeneration measured in urine. These markers of renal fibrosis have been associated with progression of CKD as measured by eGFR. However, no marker is able to predict CKD before being detected with traditional clinical tests (serum creatinine, and cystatin C, urine albumin, and ultrasound scanning) consistently and accurately. New biomarkers point to generalized processes that cause fibrosis, but they do not directly reflect kidney pathology. Biomarkers measured in serum or urine from CKD patients may not reflect the degree of kidney fibrosis and these should be corroborated with actual pathological measures of the kidney fibrosis in order to predict a given patient’s outcome.

In the context of quantification of fibrosis, several different techniques such as renal biopsy, magnetic resonance imaging (MRI), ultrasound (US) scanning can be utilized ^14, 15^. However, there are some limitations when it comes to renal biopsy as it does not cover the entire organ. The extent of fibrosis can be quantified by renal biopsy but it is an invasive procedure with associated risks ^16^.

MRI is ideally suited to quantify fibrosis via late Gadolinium enhancement and relaxation time measurement ^14, 15, 17^. Previously, we have shown that young kidney patients on maintenance dialysis develop myocardial fibrosis quantifiable via MRI T2 relaxation time ^15^. But MRI is an expensive modality, which is not readily accessible to the general population at risk of CKDu in Sri Lanka.

Fibrosis decreases the elasticity of tissue, hence measures of tissue elasticity is a surrogate marker of fibrosis. There are several techniques to measure elasticity in tissue using US. Among them transient elastography is the most common and widely used method. It measures tissue deformation while applying external pressure to the organ. Due to its retroperitoneal position this is not feasible in the kidney though it has been successfully used to quantitate liver fibrosis.

Acoustic radiation force impulse (ARFI) imaging and shear wave velocity (SWV) are two other US elastography techniques. However, the reliability of these techniques in measuring kidney fibrosis has not been consistent ^18– 20^. Recent feasibility studies show that both ARFI and SWV failed to correlate with kidney fibrosis ^19, 20^. Unlike liver, the kidney is not homogenous in tissue character; it is more perfused, with two distinct zones and pathologically more complex. Therefore in our opinion, and as evident by these studies, elasticity of the kidney is probably a less reliable surrogate for kidney fibrosis.

## Hypothesis

Our approach to developing a non-invasive imaging method to detect early signs of CKD is based on the following hypothesis:

Disease severity in CKD is associated with changes in the Fourier transform of the scattered ultrasound waves (Radio Frequency Spectrum) from the cortex of the CKD kidney.

The rationale for our hypotheses is based on the ultrasound physics.

The speckle patterns in B-Mode ultrasound images is the result of interference of scattered ultrasound waves from scatterers whose size is much smaller than the ultrasound wave length. B-mode ultrasound images are constructed from the amplitude modulation of the time domain scattered signal, known as the Radio Frequency echo (RF echo). In B-mode ultrasound the frequency dependent information of the RF echo is not utilized. However, theoretical and phantom studies ^21^ have shown that the frequency spectrum of the RF echo (RF spectrum), can be related to microstructural properties such as shape, size, density and acoustical properties of tissue. Thus, changes in scatterer properties in tissue may affect the RF spectrum. On the other hand, due to its size and structure, accumulation of fibrosis could change the scatterer properties of tissue. Thus, it is plausible that increasing fibrosis in the CKD kidney, and therefore the severity of the disease, is associated with changes in the RF spectrum. This hypothesis is reinforced by studies in the liver showing correlation between B-mode ultrasound features and the presence of fibrosis ^22– 24^.

A simple ultrasound system capable of RF echo signal acquisition and spectral analysis will be constructed to perform *ex vivo* and clinical studies. In the *ex vivo* experiment, correlations between the RF spectrum, speckle patterns and tissue characteristics will be investigated. In the clinical study, correlations between the RF spectrum, speckle pattern and the CKD stage will be investigated. In both cases speckle patterns will be used as the link between RF spectrum and fibrosis because speckle patterns are known to correlate with fibrotic stage in the liver ^22– 24^.

## The portable US system

Majority of the affected population consist of rural farmers who cannot afford to undergo regular medical screenings. Here we present an affordable and portable ultrasound system with the proposed technology that can be taken to the people at risk, where they live.

**Figure 1.**
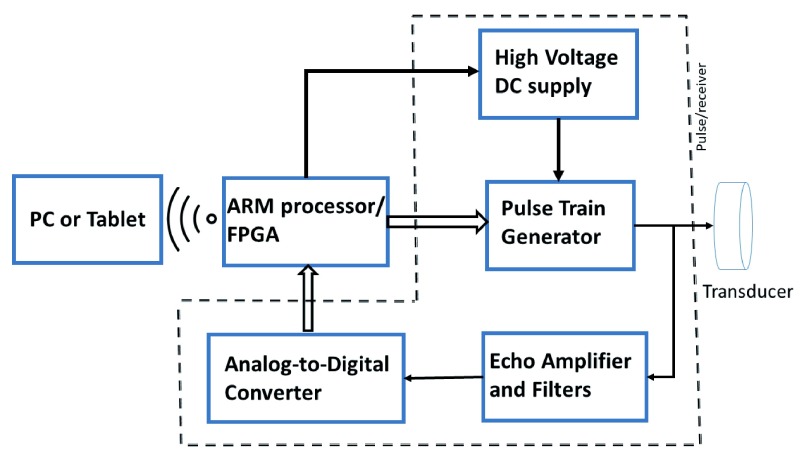
Block diagram of portable ultrasound device. The proposed system will have five major components: Pulse train generator, high voltage amplifiers, A controlling unit consist of a Field Programmable Gate Array (FPGA) and an ARM microcontroller, ultrasound transducer, and an analog-to-digital converter.

The proposed portable ultrasound probe system will have five major components. They are: waveform generator, high voltage amplifiers, a controlling unit consist of a Field Programmable Gate Array (FPGA) and an ARM microcontroller, ultrasound transducer, and an analog-to-digital converter. A block diagram of the system is shown in Figure 1.

The controller will initiate a series of 1 – 10 MHz high voltage pulses through the ultrasonic transducer. The echo received by the same transducer, after suitable amplification and filtering will be digitized at the rate of 125 MS/s. This data will be processed by the microcontroller and then will be transmitted to a laptop computer via Wi-Fi for further analysis and display.

A single element immersion transducer (Olympus V310-SU 5 MHz and V312-SU 10 MHz) will be used. Data analysis software will be developed and installed on the PC.

It is estimated that a prototype of the proposed US system can be built for approximately USD 1400.

## Proposed *ex vivo* experiment

In the laboratory experiment, RF spectrum and speckle pattern analysis will be performed on a range of *ex vivo* tissue (e.g. bovine liver, kidney tissue, etc.). The tissue sample will be completely submerged in degassed water (0.9% saline solution) and a 5 MHz or 10 MHz single element 0.25 in elemental diameter; immersion transducer (Olympus-V310-SU, Band width 16–24 MHz at -6 dB) will be used for scanning. Tissue sample will be held stationary and the transducer will move 200-micrometer step using computer controlled micro position system. RF echo data will be acquired using the proposed US system. A Hamming window will be applied to RF signal followed by fast Fourier transform (FFT) to calculate the RF power spectrum ^21^. The RF spectrum will be normalized by a spectrum obtained from a standard phantom with identical acquisition parameters. The calibrated power spectrum will be fitted to a linear model over its bandwidth. This will give the standard spectral parameters: spectral intercept (dB; extrapolation to zero frequency) and spectral slope. In addition to the standard spectral parameters we will explore other statistical measures of the spectrum in order to identify attributes of the RF spectrum that are most sensitive to tissue characteristics.

The RF data from different *ex vivo* samples will be used to construct a B-Mode ultrasound image of the sample. This image will be analyzed for speckle features employing standard techniques such as statistical methods, model-based approaches, signal processing and geometrical analysis ^25^. The speckle feature parameters will be tested on their sensitivity to differentiate *ex vivo* tissue types. The best performing speckle feature parameters will be analyzed to find correlations with RF spectrum parameters.

To assess the sensitivity and the specificity of the US system, histopathology data of human kidneys will be used. Autopsied human kidneys with different level of CKD will be collected form the pathologists. Kidney fibrosis will be determined by calculating the percentage of Masson’s Trichrome stained area of interstitial fibrosis per total area of kidney tissue. The stained areas will be analyzed with available image software, and the levels of lesion formation will be expressed as percent lesion area per total area. Kidney samples will be scanned using the immersion US probe. Back scattered US signal will be collected and analyzed to determine the sensitivity of RF and speckle feature parameters to different levels of kidney fibrosis in the autopsied human kidneys.

## Proposed clinical study

In the clinical study, ultrasound imaging will be performed on human subjects with approval from the institutional ethical committee. The standard diagnostic criteria will be applied to diagnose CKD participants and they will be classified into stages (five) according to eGFR by using CKD-EPI equation ^26^. The sample size will be based on the uncertainty of textural parameters determined in the experimental work.

B-mode ultrasound imaging will be performed with standard imaging equipment to depict long-axis and transverse views of the kidneys. Kidney longitudinal length (Bi-parietal) will be measured for both kidneys. Additionally, kidney size, cortical echogenicity, parenchymal thickness and cortico-medullary demarcation will be recorded. All clinical results will be normalized to anthropometric features such as body height etc. ^27^. The proposed US probe will be used to acquire RF echo data. The RF echo data will be transmitted to a remote server where the RF spectrum will be analyzed to quantitate RF spectrum parameters found in the laboratory experiment.

Anonymized B-mode data will be used to stratify CKD patients using speckle parameters developed in experimental work. The Spearman’s correlation test will be used to assess any correlations between the clinical and biochemical data with B-mode speckle parameters. The positive predictive values of the B-mode speckle scoring system and RF spectrum parameters will be compared with the results of the CKD stages. It is expected that the combination of *ex vivo* and clinical study results will enable us to identify an optimal set of RF spectrum parameters that will be used to diagnose early signs of CKD.

The successful completion of this project will result in, a novel ultrasound parameter of CKD that can detect the disease at early stages and technology to construct a device that can make noninvasive diagnostic measurements of the kidney. A simple diagnostic tool that is portable will have significant impact on the future studies of CKD. This ultrasound device technology that will be developed in this study is potentially patentable and has a commercial value.
