# Supplementary material for: A novel ultrasound technique to detect early chronic kidney disease
Source: F1000Res. 2019 Feb 24;7:448. Originally published 2018 Apr 11. [Version 2] doi: 10.12688/f1000research.14221.2 (PMC6415322; doi:10.12688/f1000research.14221.2)
Supplement: Peer review history

# Peer review: Reviewer response for version 2

**DOI:** 10.5256/f1000research.20082.r44855

**Published:** 2019-03-12

**Authors:** Gaetano Lucisano

**Affiliations:** 1 Imperial College Renal and Transplant Centre, London, UK

No further comments.

Is the topic of the opinion article discussed accurately in the context of the current literature?

Yes

Are arguments sufficiently supported by evidence from the published literature?

Yes

Are all factual statements correct and adequately supported by citations?

Yes

Are the conclusions drawn balanced and justified on the basis of the presented arguments?

Partly

Reviewer Expertise:

Ultrasound in Nephrology; Transplant Medicine; Transplant Immunology

I confirm that I have read this submission and believe that I have an appropriate level of expertise to confirm that it is of an acceptable scientific standard.

# Peer review: Reviewer response for version 1

**DOI:** 10.5256/f1000research.15472.r41537

**Published:** 2019-01-23

**Authors:** Federico Nalesso

**Affiliations:** 1 DIMED - UOC NEFROLOGIA 2- Azienda Ospedaliera, Università di Padova, Padova, Italy

The article introduces a new very interesting ultrasound examination to be used for the study of CKD.

 The parameters used to perform the ultrasound measurements to be correlated with renal fibrosis appear to be very difficult to measure and operator-dependent. The standardization appears difficult and very difficult to obtain. The vascular component during fibrosis is not taken into consideration and the correlation of the new ultrasound measurement with renal resistance indices is not analyzed.

 CKD is reduced to the fibrotic component only, without considering the vascular alterations that underlie interstitial changes.

Is the topic of the opinion article discussed accurately in the context of the current literature?

Partly

Are arguments sufficiently supported by evidence from the published literature?

No

Are all factual statements correct and adequately supported by citations?

Partly

Are the conclusions drawn balanced and justified on the basis of the presented arguments?

Yes

Reviewer Expertise:

CKD, AKI, US in AKI/CKD. Critical care nephrology.

I confirm that I have read this submission and believe that I have an appropriate level of expertise to confirm that it is of an acceptable scientific standard.

# Peer review: Reviewer response for version 1

**DOI:** 10.5256/f1000research.15472.r41738

**Published:** 2019-01-10

**Authors:** Gaetano Lucisano

**Affiliations:** 1 Imperial College Renal and Transplant Centre, London, UK

This is the proposal of a prospective study aimed to assess the ability of a novel portable ultrasound (US) system to estimate the amount of renal fibrosis. The study would first consist in an ex-vivo experiment in which the US system is tested on bovine solid organs (kidney and liver), ultimately leading to the clinical study on human subjects. In the first instance, the novel US system would be employed as a screening tool for the early diagnosis and staging of chronic kidney disease in the rural population of Sri Lanka.

 The idea and purposes are interesting. Few comments:

1. It is not clear whether the data obtained ex-vivo would be compared against histopathology data, as this would be imperative before proceeding with the clinical study. In this case the investigators should make sure that bovine kidneys with different degrees of fibrosis are analysed in order to properly assess the sensitivity and specificity of the US system.
2. It would be interesting to have an estimate of the saved costs of this technique employed for the rural population of Sri Lanka, as the application could be extended to the many other populations across the world with limited access to the healthcare system.
3. In my view, it is important to point out that, if successful, this US system is aimed to flag patients with suspected CKD - who will eventually need a biochemical estimation of the degree of CKD, as I doubt this system would have sufficient sensitivity to warrant a fairly accurate monitoring of the progression of the CKD (changes in the degree of fibrosis detected by the US system would probably correspond to dramatic decreases of the eGFR).
4. In the clinical study, the obtained B-mode parameters should be corrected for anthropometric features, such as the body height. This would strengthen the degree of correlation between CKD stage and US parameters (Lucisano *et al.,* 2015 ^1^).

Is the topic of the opinion article discussed accurately in the context of the current literature?

Yes

Are arguments sufficiently supported by evidence from the published literature?

Yes

Are all factual statements correct and adequately supported by citations?

Yes

Are the conclusions drawn balanced and justified on the basis of the presented arguments?

Partly

Reviewer Expertise:

Ultrasound in Nephrology; Transplant Medicine; Transplant Immunology

I confirm that I have read this submission and believe that I have an appropriate level of expertise to confirm that it is of an acceptable scientific standard, however I have significant reservations, as outlined above.

## References

[1] : Can renal sonography be a reliable diagnostic tool in the assessment of chronic kidney disease?. *J Ultrasound Med*.2015;34(2) : 10.7863/ultra.34.2.299 299-306 10.7863/ultra.34.2.299 25614403

# Response

**Published:** 2019-02-19

**Authors:** Janaka Wansapura

**Affiliations:** University of Colombo, Sri Lanka

**Response to reviewer:**

**1) It is not clear whether the data obtained ex-vivo would be compared against histopathology data, as this would be imperative before proceeding with the clinical study. In this case the investigators should make sure that bovine kidneys with different degrees of fibrosis are analysed in order to properly assess the sensitivity and specificity of the US system.**

We agree with the reviewer’s concerns and have now included a section for sensitivity analysis as follows:

To assess the sensitivity and the specificity of the US system, histopathology data of human kidneys will be used. Autopsied human kidneys with different level of CKD will be collected form the pathologists. Kidney fibrosis will be determined by calculating the percentage of Masson’s Trichrome stained area of interstitial fibrosis per total area of kidney tissue. The stained areas will be analyzed with available image software, and the levels of lesion formation will be expressed as percent lesion area per total area. Kidney samples will be scanned using the immersion US probe. Back scattered US signal will be collected and analyzed to determine the sensitivity of RF and speckle feature parameters to different levels of kidney fibrosis in the autopsied human kidneys.

**2) It would be interesting to have an estimate of the saved costs of this technique employed for the rural population of Sri Lanka, as the application could be extended to the many other populations across the world with limited access to the healthcare system.**

The following text was added:

It is estimated that a prototype of the proposed US system can be built for approximately USD 1400.

**3) In my view, it is important to point out that, if successful, this US system is aimed to flag patients with suspected CKD - who will eventually need a biochemical estimation of the degree of CKD, as I doubt this system would have sufficient sensitivity to warrant a fairly accurate monitoring of the progression of the CKD (changes in the degree of fibrosis detected by the US system would probably correspond to dramatic decreases of the eGFR)**

We understand the reviewer’s concern regarding the sensitivity of the proposed US system. The proposed clinical study, by way of testing the hypothesis of this paper, is only aimed at establishing a novel set of US based markers for kidney fibrosis. To study the sensitivity and specificity of these makers in relation to CKD, a rigorous clinical study must be performed and the data must be corroborated with established markers such as eGFR. We think such a study is beyond the scope of this hypothesis paper.

**4) In the clinical study, the obtained B-mode parameters should be corrected for anthropometric features, such as the body height. This would strengthen the degree of correlation between CKD stage and US parameters (Lucisano *et al.,* 2015** **^1^** **).**

As proposed in this study, both ex vivo and clinical study results will be used to identify optimal set of RF spectrum parameters that will lead to identifying CKD at early stages. As Lucisano *et al.* observed, all clinical results such as one-axis and transverse views of the kidneys, Kidney longitudinal length (Bi-parietal), kidney size, cortical echogenicity, parenchymal thickness and cortico-medullary demarcation, etc. will be corrected for anthropometric features such as body height etc.

The following text was added to the manuscript:

All clinical results will be normalized for anthropometric features such as body height etc. (27).
